# The Aging, Community and Health Research Unit Community Partnership Program (ACHRU-CPP) for older adults with diabetes and multiple chronic conditions: study protocol for a randomized controlled trial

**DOI:** 10.1186/s12877-021-02651-7

**Published:** 2022-02-04

**Authors:** Jenny Ploeg, Maureen Markle-Reid, Ruta Valaitis, Kathryn Fisher, Rebecca Ganann, Johanne Blais, Tracey Chambers, Robyn Connors, Andrea Gruneir, France Légaré, Janet MacIntyre, William Montelpare, Jean-Sébastien Paquette, Marie-Eve Poitras, Angela Riveroll, Marie-Lee Yous, on behalf of the ACHRU-CPP Research Team, Jenny Ploeg, Maureen Markle-Reid, Ruta Valaitis, Kathryn Fisher, Rebecca Ganann, Johanne Blais, Andrea Gruneir, France Légaré, Janet MacIntyre, William Montelpare, Jean-Sébastien Paquette, Marie-Eve Poitras, Angela Riveroll, Ali Ben Charif, Dean Eurich, Amiram Gafni, Gary Lewis, Lynne Mansell, Janet Pritchard, Diana Sherifali, Lehana Thabane, Ross Upshur, Tyler Williamson, Melissa Northwood, Cheryl Sadowski, Frank Tang

**Affiliations:** 1 School of Nursing, Aging, Community and Health Research Unit, Faculty of Health Sciences, McMaster University, 1280 Main Street West, Room HSc3N25, Hamilton, Ontario L8S 4K1 Canada; 2 Department of Family Medicine and Emergency Medicine, Faculty of Medicine, Université Laval, Pavillon Ferdinand-Vandry, 1050, avenue de la Médecine, Local 4617, Québec, G1V 0A6 Canada; 3 Department of Applied Human Sciences, Faculty of Science, University of Prince Edward Island, Room 111, Steel Building, 550 University Avenue, Charlottetown, Prince Edward Island C1A 4P3 Canada; 4 Department of Family Medicine Research Program, University of Alberta, 6-40 University Terrace, Edmonton, Alberta T6G 2T4 Canada; 5 VITAM-Centre de recherche en santé durable, Université Laval, Pavillon Landry-Poulin, 2525, Chemin de la Canardière, Quebec City, QC, G1J 0A4, Canada and Department of Family Medicine and Emergency Medicine, Faculty of Medicine, Université Laval, Québec, G1K 7P4 Canada; 6 Faculty of Nursing, Room 116, Health Sciences Building, University of Prince Edward Island, 550 University Avenue, Charlottetown, Prince Edward Island C1A 4P3 Canada; 7 Margaret and Wallace McCain Chair in Human Development and Health, Department of Applied Human Sciences, Faculty of Science, Room 122, Health Sciences Building, University of Prince Edward Island, 550 University Avenue, Charlottetown, Prince Edward Island C1A 4P3 Canada; 8 Groupe de Médecine de Famile Universitaire (GMF-U) du Nord de Lanaudière and Department of Family Medicine and Emergency Medicine, Faculty of Medicine Université Laval, Pavillon Ferdinand-Vandry, 1050, Avenue de la Médecine, Local 4617, Québec, G1V 0A6 Canada; 9 Department of Family Medicine and Emergency Medicine, Faculty of Medicine and Health Sciences, Université de Sherbrooke - Campus Saguenay, 305 Rue Saint Vallier, Chicoutimi, QC, G7H 5H6 Canada; 10 Department of Applied Human Sciences, Faculty of Science, University of Prince Edward Island, Room 115, Steel Building, 550 University Avenue, Charlottetown, Prince Edward Island C1A 4P3 Canada

**Keywords:** Older adults, Patient-oriented intervention, Diabetes, Multiple chronic conditions, Pragmatic effectiveness-implementation trial, Self-management, Community-based settings, Scalability assessment

## Abstract

**Background:**

Older adults (≥65 years) with diabetes and multiple chronic conditions (MCC) (> 2 chronic conditions) experience reduced function and quality of life, increased health service use, and high mortality. Many community-based self-management interventions have been developed for this group, however the evidence for their effectiveness is limited. This paper presents the protocol for a randomized controlled trial (RCT) comparing the effectiveness and implementation of the Aging, Community and Health Research Unit-Community Partnership Program (ACHRU-CPP) to usual care in older adults with diabetes and MCC and their caregivers.

**Methods:**

We will conduct a cross-jurisdictional, multi-site implementation-effectiveness type II hybrid RCT. Eligibility criteria are: ≥65 years, diabetes diagnosis (Type 1 or 2) and at least one other chronic condition, and enrolled in a primary care or diabetes education program. Participants will be randomly assigned to the intervention (ACHRU-CPP) or control arm (1:1 ratio). The intervention arm consists of home/telephone visits, monthly group wellness sessions, multidisciplinary case conferences, and system navigation support. It will be delivered by registered nurses and registered dietitians/nutritionists from participating primary care or diabetes education programs and program coordinators from community-based organizations. The control arm consists of usual care provided by the primary care setting or diabetes education program. The primary outcome is the change from baseline to 6 months in mental functioning. Secondary outcomes will include, for example, the change from baseline to 6 months in physical functioning, diabetes self-management, depressive symptoms, and cost of use of healthcare services. Analysis of covariance (ANCOVA) models will be used to analyze all outcomes, with intention-to-treat analysis using multiple imputation to address missing data. Descriptive and qualitative data from older adults, caregivers and intervention teams will be used to examine intervention implementation, site-specific adaptations, and scalability potential.

**Discussion:**

An interprofessional intervention supporting self-management may be effective in improving health outcomes and client/caregiver experience and reducing service use and costs in this complex population. This pragmatic trial includes a scalability assessment which considers a range of effectiveness and implementation criteria to inform the future scale-up of the ACHRU-CPP.

**Trial registration:**

Clinical Trials.gov Identifier NCT03664583. Registration date: September 10, 2018.

**Supplementary Information:**

The online version contains supplementary material available at 10.1186/s12877-021-02651-7.

## Background

Diabetes is a common chronic condition in developed countries and its prevalence is rising [1]. Older adults have the highest prevalence of diabetes of any age group [2] and comorbidity is common, with upwards of 40% of clients with diabetes having three or more comorbidities [1, 3]. Persons with diabetes who have higher comorbidity burden face additional care challenges that result in higher levels of functional impairment, reduced quality of life, increased health service use, and higher mortality [2, 4]. Community-living older adults with multiple chronic conditions (MCC) also rely heavily on family caregivers [5]. In 2018, approximately 25% of Canadians aged 15 years and older (7.8 million) provided care to a family member or friend with a long-term health condition, physical or mental disability or issue related to aging [6]. Caregivers experience negative impacts of caregiving including poorer mental and physical health and financial costs [7]. Caregivers report that their needs are not addressed [8], potentially resulting in increased use of acute care services (e.g., hospitalization, emergency department (ED) visits) [9].

Given the high rates of comorbidity among older adults with diabetes, research and models of care require a shift from a single-disease paradigm to a MCC framework and one that considers the range of individual, social and system factors shaping health [10]. Chronic disease rates are often higher among those who have low incomes, limited education, live in remote regions, or face social disadvantages [11], suggesting that programs benefitting clients must reduce barriers across service providers, organizations, and sectors. Recent systematic reviews suggest that integrated case management and care continuity result in reduced ED and acute care service use [12, 13]. Research on older adults with MCC also suggests that programs need to be person-centred, multifaceted, tailored to individual needs, and engage clients and caregivers in co-designing, implementing, and evaluating programs [14, 15]. To maximize client benefit and achieve population-wide impact, assessing patient-oriented outcomes (e.g., quality of life, physical function, mental health) and identifying barriers and facilitators to program implementation in diverse populations and contexts are recommended.

Self-management interventions, such as those in the American Diabetes Prevention Program [16] and Finnish Diabetes Prevention Study [17], are also recommended because they demonstrate that lifestyle changes are effective and have sustainable benefits [18–23]. These interventions appear to target constructs of the social cognitive theory that support behaviour change, such as observational learning, reinforcement and expectations within a social context, and self-efficacy [24, 25]. Most older adults with diabetes do not meet physical activity recommendations, with social cognitive theory constructs explaining almost half of the variance in participation in moderate physical activity for this group [26]. An integrative review found that supporting self-care in older adults with diabetes using interventions that integrate concepts of self-efficacy, self-determination and proactive coping were effective in influencing diabetes self-care behaviours (e.g., exercise, dietary control) and health outcomes (e.g., glycated hemoglobin [HbA1c]) [27]. A systematic review of self-management programs for older adults with diabetes found that interventions that were tailored (i.e., customized to client needs and goals) or emphasized psychological support (i.e., focused on distress, depression or coping) were the most effective at reducing HbA1c levels (-0.20, 95% CI: -0.40 to -0.10) [28].

Despite the evidence supporting tailored self-management interventions that target a broad range of health determinants, there remains limited rigorous evidence for the effectiveness of community-based self-management programs for older adults with diabetes and MCC, due to the exclusion of this complex group from most trials [29]. Further, few studies evaluate non-clinical measures that are important to clients, integrate caregivers in care, examine costs of health service use using a comprehensive and societal perspective, or involve programs that link primary care with other community-based services to improve access and address the full range of social determinants of health. Even fewer studies consider key post-study issues in the design and evaluation phase, such as sustainability of the intervention once the clinical trial has ended and scaling-up interventions that are proven to be effective. Consideration of these issues in turn requires a strategy rarely employed in clinical trials to date, which is engaging clients and their caregivers, as well as providers and decision-makers, as research partners in the planning, development, implementation, and evaluation of the intervention.

The proposed research study aims to address these deficiencies in the current evidence base and the design of clinical trials involving the target population. Our goal is to design and deliver an intervention that meets the complex and varied needs of older adults with diabetes and MCC, while producing the evidence on effectiveness as well as how the intervention should be implemented on a larger scale. This study focuses on evaluating and scaling up the Aging, Community and Health Research Unit Community Partnership Program (ACHRU-CPP), an intervention that was co-designed by clients, caregivers, primary care providers and researchers [30]. This self-management intervention for older adults with diabetes and MCC involves integration of primary care and community care services and includes a focus on the broader determinants of health affecting older adults and their caregivers. The intervention has been tested in two Canadian provinces (Ontario and Alberta) and selected primary care and community settings [31, 32]. Our intent with the proposed study is to add to the existing evidence base for the intervention by testing it in a broader range of socio-cultural, practice and jurisdictional settings within Canada. Compared to our previous trials, this trial will attempt to recruit a higher proportion of vulnerable subgroups. We will also incorporate scale-up planning into this study’s design and implementation, to address a recognized gap in the existing literature. A recent systematic review on scaling-up primary care interventions noted vast inconsistencies in scaling-up reports, with none reporting information on assessing scalability or the effectiveness of scale-up strategies [33]. Accordingly, we will respond to the advice from experts to use an incremental approach to scale-up where ongoing monitoring of implementation and effectiveness is done to build the evidence base [34].

## Research aim, hypotheses and questions

The trial aims to evaluate the effectiveness and implementation of the ACHRU-CPP compared to usual care. We hypothesize that clients in the intervention compared to usual care group will experience greater improvements in mental and physical functioning and a range of other outcomes linked to health benefits (e.g., improved diabetes self-management, increased physical activity, reduced nutrition risk). We also expect that the intervention will be cost neutral relative to usual care because the intervention costs will be offset by lower use of other costly acute care services (e.g., hospitalization, ED use). Finally, we expect that the intervention will have potential for scale-up.

The study will address the following research questions regarding the six-month self-management intervention:

1. What is the effect of the intervention compared to usual care on mental functioning (primary outcome) and physical functioning, diabetes self-management, depressive symptoms, anxiety, social support, physical activity, activities of daily living/instrumental activities of daily living, nutrition risk, food security, and costs of use of health services (secondary outcomes) in older adults aged ≥ 65 years with diabetes and one or more chronic conditions?
2. What is the effect of the intervention compared to usual care on outcomes (health-related quality of life, depressive symptoms, anxiety, caregiver strain, costs of use of health services) of family and friend caregivers aged ≥18 years?
3. Which subgroups of older adults (e.g., males/females, those with more/less chronic conditions) benefit most from the intervention?
4. How is the intervention adapted and implemented in diverse settings?
5. What is the scalability of the intervention?
6. What are the experiences of older adults and caregivers who engage in the research process?

In this study, we will recruit participants and their caregivers from geographic areas that include populations who: have low socioeconomic status, are multicultural, live in urban and rural areas, and have French and English-speaking residents. We will conduct subgroup analyses to explore which groups benefit most from the intervention (e.g., those with more chronic conditions). It is critical to test an innovation in the socio-cultural and practice settings in which it will be scaled-up [34]. Testing to date has focused on only two Canadian provinces (Ontario and Alberta) and selected primary care and community settings [31, 32] and has not included an appreciable proportion of vulnerable population segments such as high system users, who in general are a relatively small subgroup responsible for a disproportionately high amount of service use and cost [35]. There is evidence that older adults with diabetes and MCC have higher odds of frequent ED use [12, 36, 37] and international studies link high use to the following factors: female, older, racial minority status, low socio-economic status, high acuity, social isolation, rural residence, having mental health problems, having a primary care provider, low continuity of care, and high prior year ED/hospital use [12, 38–45].

## Methods

This section begins by presenting the trial methods as per the original protocol developed prior to the Coronavirus Disease 2019 (COVID-19) pandemic. Some of the methodological components for this trial were published in the study protocol for the previous randomized controlled trial (RCT) testing the same intervention, thus we briefly summarize these methods in this paper and refer to the prior protocol paper for the details [46]. We also note that the trial that this protocol pertains to began in 2019 and was underway when the pandemic hit in March 2020, which temporarily halted the trial at all sites and triggered a number of unplanned changes to the trial. *All changes to the protocol introduced in response to the extenuating circumstances arising from the pandemic have been described at the end of the Methods section (Modifications Triggered by COVID-19).*

### Study design

This study is a cross-jurisdictional, multi-site implementation-effectiveness type II hybrid RCT, where both effectiveness and implementation will be evaluated with equal emphasis [47, 48]. Current thinking regarding the evaluation of complex interventions is consistent with the hybrid nature of our trial. Complexity is now recognized as not solely the property of the intervention, but also the context (e.g., healthcare system, practice setting) within which it is implemented and the interaction between the two [49].

The effectiveness component is designed to achieve the goals of comparative effectiveness research, which include informing practice and/or policy decisions, comparing the intervention to usual care, employing patient-relevant outcome measures, and conducting the trial in settings for which the intervention is intended [50]. To achieve these pragmatic aims, the PRagmatic Explanatory Continuum Indicator Summary Version 2 (PRECIS-2) [51] was used to design the trial with our efforts focused on maximizing pragmatism across the tool’s nine domains. This pragmatic focus links to the implementation component, which is designed as a formative evaluation to ensure that the trial is efficient at each site in adapting in real time to new information arising during the trial [52]. Standards-setting organizations recommend implementation evaluations in RCTs as they inform implementation fidelity, adaptation, reach, and the contextual factors associated with variation in outcomes [53–55]. The implementation component employs process evaluation and a realist and systems perspective, approaches that are increasingly being used to inform the evaluation of complex interventions to address the research-practice gap and maximize the usefulness of the evidence for decision-making [53].

Guidelines used in preparing this protocol include: the Template for Intervention Description and Replication (TIDieR) checklist [56] to structure the description of the intervention, the CONSORT Standardized Protocol Items: Recommendations for Interventional Trials (SPIRIT) [57] to structure the protocol, CONSERVE-SPIRIT [58] to guide the reporting of modifications to the protocol due to COVID-19, and SPIRIT [57] to inform the schedule of participant enrolment, intervention, and assessments, as discussed in detail below.

### Study setting

In Canada, provincial governments have the primary responsibility for delivering healthcare services, with the result that healthcare systems and services differ across the 10 provinces. This means that assessing scale-up on a Canada-wide basis requires testing the intervention in more than one province. The trial will be conducted in two sites in each of three Canadian provinces, Ontario, Quebec and Prince Edward Island. These provinces were chosen because their provincial governments have strategic priorities related to health care services targeting: older adults, individuals with chronic conditions, and individuals who are high users of the health care system. The provinces, and the sites within them, were also chosen to capture: (a) different geographic settings (urban and rural), (b) a growing older adult population, (c) people of diverse socio-demographic and cultural backgrounds (e.g., English and French speaking, highly culturally diverse urban settings), and (d) providers demonstrating strong support for the intervention and having staff to support its implementation.

Each site will involve a primary care setting or diabetes education program (i.e., outpatient service that promotes health and supports lifestyle changes for people with diabetes or pre-diabetes) and a community partner site (e.g., Young Men’s Christian Association [YMCA]). The community partners were selected based on proximity to the primary care setting or diabetes education program and availability of staff members to participate in delivery of the intervention (e.g., a program coordinator for the group wellness sessions).

### Study governance structures

Governance structures will be established to ensure active and ongoing stakeholder involvement in the design, implementation and evaluation of the intervention. Two study governance structures will facilitate successful intervention implementation and scalability assessment through active engagement of clients/caregivers, service providers and policy-makers: community advisory boards (CABs) and the Steering Committee. CABs will be established in local sites and will consist of clients/caregivers (excluding trial participants); service providers/managers (e.g., primary care, home and community care, public health, diabetes education program, and social services); and policy makers from regional/municipal governments. These boards will meet regularly to share information related to community assets and gaps to inform local intervention adaptations and scale-up and engage in knowledge translation activities, such as interpreting research results and crafting and disseminating key messages through their networks. The Steering Committee will consist of: policy knowledge users (e.g., from provincial health branches such as primary care, chronic disease); representatives from Diabetes Action Canada, a Strategic Patient Oriented Network in Diabetes and its Related Complications; public and community research partners; and research team members. The Steering Committee will provide guidance on: all research directions; adapting and contextualizing the intervention so it can be implemented and embedded into diverse real-world primary and community-based settings; assessing scalability; and developing and disseminating knowledge translation products.

### Eligibility criteria and recruitment procedure

There are four distinct study populations involved in the trial: (a) older adults who will receive the intervention in addition to usual care versus usual care alone; (b) family or friend caregivers of the older adults receiving the intervention, (c) providers and managers who are delivering the intervention, and (d) public and community research partners who are not receiving the intervention but are participating on governance structures (i.e., CABs and Steering Committee).

#### Older adults

Older adults will be eligible if they meet the following inclusion criteria:

i. aged ≥ 65 years;
ii. diagnosed with type 1 or type 2 diabetes;
iii. diagnosed with at least one of the following other chronic conditions (in addition to diabetes): hypertension, depression and/or anxiety, chronic musculoskeletal conditions, arthritis or rheumatoid arthritis, osteoporosis, asthma or chronic obstructive pulmonary disease or chronic bronchitis, cardiovascular disease, heart failure, stroke or transient ischemic attack, stomach problems such as reflux or gastric ulcer, colon problem, chronic hepatitis, thyroid disorder, cancer within the last five years, kidney disease or failure, chronic urinary problem, dementia or Alzheimer’s disease, hyperlipidemia, human immunodeficiency virus) [59];
iv. enrolled in a primary care setting or diabetes education program;
v. residing in the community and within the area served by the primary care setting or diabetes education program and not planning to move out of this community in the next 6 months,
vi. capable of providing informed consent, or has a substitute decision-maker (SDM) who is able to provide informed consent on his/her behalf; and
vii. competent in English or French or has an interpreter who is competent in English or French.

The Research Assistant (RA) will proceed with the completion of baseline interviews and questionnaires once the client’s (or the substitute decision-maker’s) cognitive status has been confirmed and informed consent has been obtained, as per the methods described in our previous protocol paper [46]. The RA will repeat the short portable mental status questionnaire (SPMSQ [60]) with the client or their SDM prior to completing the 6-month (T2) questionnaire. A SDM acting on behalf of an enrolled client can also be enrolled as a caregiver in the study.

#### Caregivers

Eligible caregivers are those who at the time of recruitment of the older adult are identified by the older adult as a family or friend caregiver aged ≥18 years who provides physical, emotional, or financial care to the older adult. All interviews with eligible caregivers will be conducted by telephone. At the initial home/telephone visit, the RA will administer the SPMSQ [60] prior to obtaining their oral consent to participate. If the caregiver scores less than 5 on the SPMSQ then the caregiver will not be included in the study (thus the interview will not proceed). The oral consent process will be audio-recorded prior to initiating the baseline (T1) questionnaire. The RA will repeat the SPMSQ with the caregiver, prior to completing their 6-month (T2) questionnaire.

#### Intervention providers and managers

Intervention team members including a registered nurse (RN), a registered dietitian (RD)/nutritionist, and a community program coordinator (PC) from each site, and their managers are eligible to participate in the study. The managers at the primary care site will recruit and select the members of the intervention team. To avoid contamination with the control arm, all providers that are a member of the intervention team will not deliver usual care to participants in the control arm of the study.

#### Public and community research partners

Public and community research partners include older adults and caregivers with lived experience with diabetes, local service providers and administrators, and policy- and decision-makers at the local, provincial and national level.

### Data collection

#### Participant flow, assessments, and timeline

**Fig. 1:**
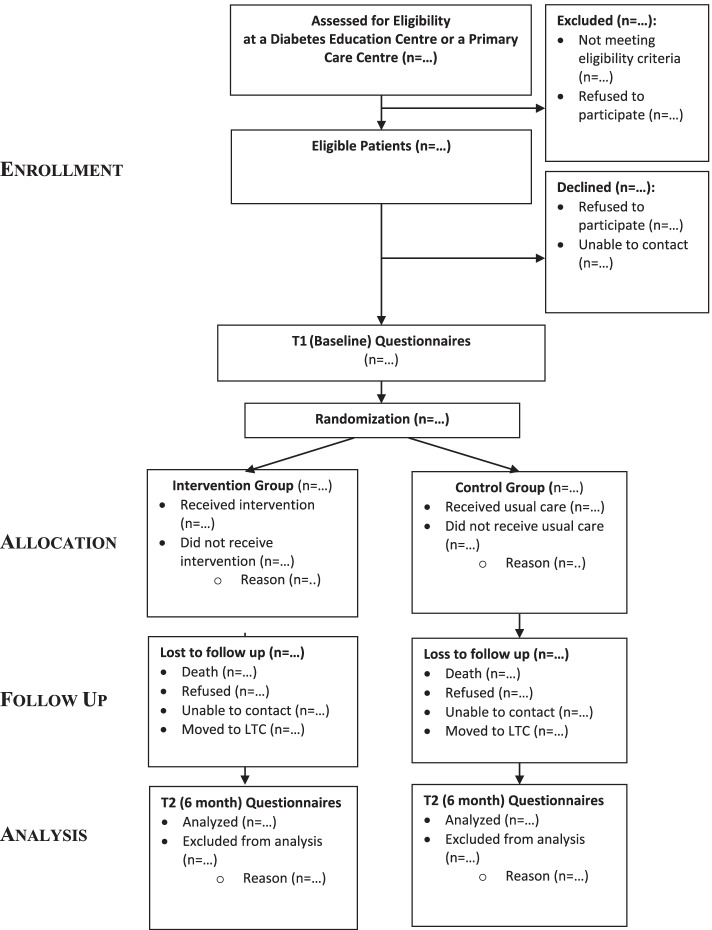
Flow Diagram of Progress through Study Phases

RAs will conduct two assessments (home or telephone interview) with each study participant, one at baseline (T1) and the other at the end of the 6-month intervention period, 6 months after baseline (T2). Baseline data will be collected on socio-demographic, clinical, and primary and secondary outcome variables. Each assessment will take approximately 1.5 hours to complete. The Research Coordinator (RC) will meet regularly with the RAs to address questions and concerns and ensure consistency of approach across the sites. The RC will check the data throughout the data collection process to ensure minimal errors and missing data. Participant flow through the study will be illustrated using a flow diagram which conforms to the CONSORT guidelines [61] for reporting pragmatic trials (See Fig. 1).

#### Sample size

The target total sample size is 264 older adults based on an effect size of 0.38 for mental functioning (primary outcome) as measured by the Mental Component Summary (MCS) score from the 12-item Medical Outcomes Study Short-Form Health Survey (SF-12) that was observed in the previous Ontario randomized controlled trial (MD = 3.68, SD=9.68, power=80%, two-sided alpha=0.05, 20% attrition) [31]. SAS Version 9.4 (PROC POWER, two-sample t-test for mean difference, equal variance, same sample size per group) was used to calculate the sample size. A total sample size of 264 will require 88 participants per province (44 participants per site).

#### Recruitment and retention

Staff from each of the primary care or diabetes education program sites will be trained to identify potential clients using existing medical records or electronic databases (e.g., electronic medical records) based on the study inclusion criteria. They will then contact potential clients by telephone to determine their interest in learning more about the study and use a standardized script to introduce the clients to the study and screen for study eligibility. Recruitment of vulnerable older adults who have multiple chronic conditions has been challenging in our own research and that of others [62, 63], thus staff that are recruiting at each site will use a clear communication strategy to share information with potential participants related to the study purpose, benefits and risks, and what is being asked of them. Eligible clients who agree to be contacted will then receive a telephone call from a study RA who will share information about the study and arrange a time for the baseline assessment. Written or audio-recorded oral informed consent will be obtained by the RA at the baseline assessment. At the baseline assessment, clients will be asked to invite their family or friend caregiver (aged ≥18 years who provides physical, emotional, or financial care) to participate in the study and provide their contact information. While participation of caregivers is encouraged, it is not required.

Those who agree to participate in the study may be more receptive to the intervention, creating sampling bias. The extent of non-response bias will be assessed by comparing baseline characteristics of those who agree and those who decline to participate in the study.

In relation to retention of study participants, the RC will mail a reminder letter to the participant three months prior to the T2 interview to remind them of the date and time of the interview with the RA. The RA will call the participant three business days prior to their T2 interview to confirm their interview time. At the participant’s request, the RA will also call the participant the day before the interview.

#### Randomization design

Randomization procedures will be carried out as described in our previous protocol paper [46]. Briefly, the procedures include random assignment to the groups using a 1:1 allocation ratio, stratified permuted block randomization with the sequence generated by a biostatistician not involved in the recruitment process and with the study sites used as strata, and sequences entered into a centralized web-based randomization service (REDCap Version 11.1.9).

#### Blinding

Participants will be blinded to their group allocation. Efforts will be made to blind the RAs who conduct the T1 and T2 interviews to group allocation, although this may be difficult to maintain in all cases (e.g., if a client refers to a home/telephone visit or group wellness education session during the interview). The statistician/data analyst will be blinded to group allocation of the participants. The intervention will be known to the providers who are administering the intervention however, they will be unaware of the outcomes being studied. Upon completion of the study, participants will receive a debriefing letter describing the two groups and their group allocation.

### Intervention arm

Key features of the intervention and control arms of the study are outlined in Additional File 2 of our previous protocol paper [46], with further details provided below.

#### Theoretical foundation

In developing complex interventions, it is important to ground them in theories that support effectiveness [53]. Bandura’s social cognitive theory [24, 25] underpins the ACHRU-CPP, consistent with many diabetes prevention/treatment interventions. The previous protocol paper discussed how the constructs in this theory relate to the principles and components of the intervention tested in this trial [46].

#### Intervention development and testing

The intervention was developed in collaboration with older adults with diabetes, caregivers, providers, and researchers [30]. The feasibility, acceptability, and preliminary effectiveness of the ACHRU-CPP were established through a feasibility study conducted with a diabetes education program in a primary care setting in partnership with a community seniors’ centre [30]. The ACHRU-CPP was adapted based on client and provider feedback, implemented and tested in a pragmatic RCT in four sites in Ontario [31] and three in Alberta, Canada [32].

#### ACHRU-CPP intervention description

Participants randomly assigned to the intervention group will be offered the 6-month intervention in addition to usual healthcare services offered by their primary care setting or local diabetes education program. The intervention will be delivered by an interprofessional team of providers in each site including RNs, RDs/Nutritionists, and a PC from a community partner organization, who may be a kinesiologist or exercise specialist.

The core components of the intervention include: (a) up to three home/telephone visits by the RN and/or the RD/Nutritionist; (b) up to six in-person monthly group wellness sessions that include health education, exercise (gentle progressive physical activity) and informal peer support; (c) ongoing nurse-led care coordination and system navigation to link clients to other health care professionals and community services as needed; (d) ongoing caregiver engagement and support during the home/telephone visits and group wellness sessions; (e) monthly case conferences of the intervention team where the team will discuss and evaluate the client’s plan of care; and (f) collaboration with the primary care team and other specialists as needed. The RN or RD/Nutritionist, with the client’s consent, will complete and send alerts (e.g., medication, depressive symptoms, diabetes complications) to communicate concerns with the primary care physician or other providers.

A care plan will be developed at the beginning of the intervention. The intervention team will collaborate with the client in developing and tailoring a coordinated care plan to meet their individual needs and preferences. The care plan will be documented, outline the goals and actions that will be undertaken to achieve the goals, and a copy of the care plan will be provided to the client and all members of the intervention team. Home/telephone visits are expected to begin within two weeks of allocation to the intervention and prior to the initial group wellness session. Subsequent visits will occur at approximately three and five months, in consultation with the participant. Progress towards achieving the goals identified in the care plan will be discussed at each visit and modified as needed.

#### Intervention implementation strategies

The strategies to be used to implement and monitor the delivery of the intervention at each site were described in the prior protocol paper [46]. Briefly these include provider training, outreach meetings between researchers and the intervention team to discuss progress and address challenges, and routine updates and completion of forms documenting intervention components delivered. Compared to previous trials, implementation strategies will increase the use of online resources and virtual methods to facilitate communication across sites and provinces and address recent challenges related to the pandemic.

### Control arm: usual care

Clients who are randomly assigned to the control group will continue to be offered usual care services through their primary care setting or local diabetes education program. The specific services that comprise usual diabetes care differ within and across provinces in terms of the length and focus of educational sessions, whether classes are strongly recommended versus optional (e.g., foot care, cardiac health, diet and exercise interventions), whether home/telephone visits are offered as part of usual care, access to on-site professionals (e.g., endocrinologist, dietitian, physiotherapist, exercise specialist, pharmacist), connections with support services and community resources, and type of follow-up services available. Details of usual care provided at each site will be carefully documented.

### Effectiveness outcomes, measures, and analyses

The effectiveness of the intervention will be evaluated using several older adult and caregiver-relevant measures. We selected standardized tools and chose measures that had been validated in the target population (where possible). We briefly summarize these measures below, and Table 1 provides the details on each measure including a description, time points collected, and associated methods of analysis.

**Table 1:** Effectiveness Outcomes, Measures, Target Population, Timing and Analysis

Variable/Outcome	Measure	Study Group(s)^a^	Timing^b^	Method of Analysis
Mental functioning	Mental component summary (MCS) from SF-12 [64]	CT, CG	T_1_, T_2_	Descriptive analyses T1-T2 (Treatment Effect); ANCOVA, subgroup analyses (sex, number of chronic conditions, province)
Physical functioning	Physical Component Summary (PCS) from SF-12 [64]	CT, CG	T_1_, T_2_	Descriptive analyses T1-T2 (Treatment Effect); ANCOVA
Diabetes self care	Summary of Diabetes Self Care Activities (SDSCA) [65]	CT	T_1_, T_2_	Descriptive analyses T1-T2 (Treatment Effect); ANCOVA
Depressive symptoms	Center for Epidemiological Studies Depression Scale (CES-D-10) [66]	CT, CG	T_1_, T_2_	Descriptive analyses T1-T2 (Treatment Effect); ANCOVA
Anxiety	Generalized Anxiety Disorder 7-item Scale (GAD-7) [67]	CT, CG	T_1_, T_2_	Descriptive analyses T1-T2 (Treatment Effect); ANCOVA
Social support	Duke Social Support Index (DSSI) [68]	CT	T_1_, T_2_	Descriptive analyses T1-T2 (Treatment Effect); ANCOVA
Physical activity	Physical Activity Scale for the Elderly (PASE) [69]	CT	T_1_, T_2_	Descriptive analyses T1-T2 (Treatment Effect); ANCOVA
Activities of daily living/instrumental activities of daily living	Older Americans Resources and Services Multidimensional Functional Assessment Questionnaire Activities of Daily Living/Instrumental Activities of Daily Living (OARS ADL/IADL) [70, 71]	CT	T_1_, T_2_	Descriptive analyses T1-T2 (Treatment Effect); ANCOVA
Eating and nutrition risks	Seniors in the Community: Risk Evaluation for Eating and Nutrition (SCREEN II-AB) [72]	CT	T_1_, T_2_	Descriptive analyses T1-T2 (Treatment Effect); ANCOVA
Food security	Single item from Canadian Longitudinal Study on Aging [73]	CT	T_1_, T_2_	Descriptive analyses
Diabetes-related complications	Self-report of retinopathy, neuropathy, peripheral vascular disease	CT	T_1_, T_2_	Descriptive analyses
Laboratory values	HbA1c, e-GFR, LDL cholesterol	CT	T_1_, T_2_	Descriptive analyses, Inferential statistical analysis of T1-T2 change
Caregiver strain	Modified Caregiver Strain Index (CSI) – 13 items [74]	CG	T_1_, T_2_	Descriptive analyses T1-T2 (Treatment Effect); ANCOVA
Utilization of health and social services and associated costs	Health and Social Services Utilization Inventory (HSSUI) [75, 76]	CT, CG	T_1_, T_2_	Descriptive analyses T1-T2 (treatment Effect); Parametric or non-parametric tests depending on distributional properties of cost data, complete case analysis, Hazard Ratio (95% CI) & Risk Difference (95% CI) for ED visits and hospital admissions
Interprofessional team functioning	Partnership Self-Assessment Tool (PSAT) [77]	INT	3 months and end of intervention	Descriptive analyses
Public and community partner engagement	The Quality Involvement Measure [78] The Patient-Centred Outcomes Research Institute’s Ways of Engaging – Engagement Activity (WE-ENACT) Tool [79] Selected items of the Patient-Centred Outcomes Research Institute’s Engagement Activity Inventory (NET-ENACT) Tool [80]	Public and community research partners	3 months and end of intervention	Descriptive analyses

^a^ Four study groups: CT: client; CG: caregiver; INT: intervention team; Public and community research partners.

^b^ Two time points: T_1_: baseline; T_2_: 6 months after baseline.

^c^ PSAT will be completed by core intervention team (e.g., Registered Nurse, Registered Dietitian/Nutritionist, Program Coordinator)

#### Older adult participant outcomes and measures

The primary outcome is the mental function of the older adult participants as measured by the MCS score from the SF-12 [64]. This measure was chosen because functional status is a patient-reported outcome measure (PROM) recognized as important to a wide range of patients including those receiving self-management interventions targeting chronic conditions [81, 82]. We also found this measure to be responsive to the ACHRU-CPP in our previous trial [31] and mental functioning is linked to self-efficacy and the behaviour change constructs that play a central role in the theory underpinning the intervention. As shown in Table 1, a range of secondary outcome measures are also being assessed to capture physical functioning, activity levels, and limitations; diabetes self-management activities; various mental health outcomes; social support; nutrition risks; and clinical measures representing key diabetes risk factors such as measures of blood sugar control (HbA1c), kidney function (GFR), and cholesterol level (LDL) where available in electronic medical records.

The Health and Social Services Utilization Inventory (HSSUI) [46, 75, 76] will be used to measure the use of health and social services as reported by study participants. The six-month cost data will be derived from the product of the “quantity” data on the HSSUI and 2020-2021 “price” data obtained by our team from provincial databases. The costs of use of health and social services will include costs of intervention-specific services such as home/telephone visits and group wellness sessions.

#### Caregiver outcomes and measures

The effectiveness of the intervention on caregiver participants will be assessed on a range of outcomes including mental functioning, physical functioning, mental health, caregiver strain, and self-reported use of acute care services (EDs and hospitals) (See Table 1).

#### Analysis: effectiveness evaluation (older adult and caregiver participants)

The baseline demographic and clinical characteristics of participants who completed both the baseline and 6-month assessments will be compared. Descriptive statistical analysis will be used to summarize outcome variables at baseline and 6 months. For continuous variables, means and standard deviations will be calculated, and for categorical variables, frequencies and proportions will be assessed. Analysis of covariance (ANCOVA) will be used to test the differences in outcome variables between the intervention and control group at 6 months. Separate ANCOVA models will be run for each primary and secondary outcome, with the 6-month change in the outcome as the dependent variable, group (intervention, control) as the independent variable, and the baseline value of the outcome as the covariate.

Statistical tests will use a 0.05 two-tailed level of significance and 95% confidence levels. An intention-to-treat (ITT) approach unadjusted for baseline characteristics will represent the primary analysis, using multiple imputation for missing data. Costs will be applied to the health and social service use reported by clients and caregivers. Group differences in the 6-month change in service use costs will be examined using parametric or non-parametric methods, depending on the distributional properties of the cost data [83].

Sensitivity analyses will be conducted to test the robustness of the results for the primary outcome to various assumptions and analytical approaches used in the primary analyses [84]. A complete case analysis will be conducted to provide a comparison to the ITT results obtained using multiple imputation. A multivariable analysis that adjusts for residual baseline imbalances will be conducted to assess their impact on the effect estimates, if significant imbalances between groups exist post-randomization [85]. As a multi-site RCT, there exists the possibility that participants within a site may be more homogeneous compared to those from another site, thus a sensitivity analysis will be conducted that includes site as a covariate. Distributional assumptions underlying the ANCOVA model will be tested, with non-parametric methods explored if significant violations are present [86]. Since some participants in the intervention group were not offered the full intervention or did not receive any of the intervention (e.g., resulting from choice, due to COVID-related issues), we will conduct an analysis that excludes participants in the intervention group that did not receive any of the intervention for comparison with the results from the primary analysis (which include participants irrespective of whether they received the intervention or not). Finally, exploratory subgroup “sensitivity” analyses will be conducted to assess the potential benefit of the intervention for clinically-meaningful subgroups. The guidance from the recently-published Instrument for assessing the Credibility of Effect Modification Analyses (ICEMAN) [87] was reviewed in an effort to maximize the credibility of the subgroup analysis (if undertaken). The following three factors were pre-selected for the subgroup analysis: sex, number of chronic conditions, and province. We hypothesize that the intervention will be more effective in men because they typically engage in fewer self-management activities and access health care services less frequently than women [88]. We expect the intervention will be less effective in those with a higher number of chronic conditions because of the likelihood that they would have more complex care needs. We also expect to see variation in intervention effects across provinces due to contextual differences (e.g., health care systems, culture, rurality). Regression using two-way interactions between the study group (intervention versus control) and each characteristic will be conducted to determine subgroup effects [89, 90]. It is important to note that the subgroup “sensitivity” analyses will be regarded as exploratory, given the potential for the overall type I error to be inflated [86].

### Implementation outcomes, measures, and analyses

In this sub-section, implementation evaluation will be described, including implementation outcomes, adaptation and implementation of the intervention.

#### Implementation outcomes and measures

Implementation outcomes that will be assessed in this study are defined in Tables 2 and 3 and include adoption, acceptability, feasibility, and fidelity.

**Table 2:** Study Implementation Evaluation Summary

Question/Focus	Definition/Data Source	Method of Analysis
Implementation outcomes a) Adoption (uptake) b) Acceptability c) Feasibility d) Fidelity	a) Engagement rate b) Post-intervention interviews with clients, caregivers, intervention providers and program managers c) Recruitment rate d) Fidelity checklist	a) Proportion of intervention participants who receive at least one home/telephone/videoconference visit and attend at least one group wellness session Dose of the intervention defined as number of home/telephone/videoconference visits and number of group wellness sessions b) Qualitative content analysis c) Proportion of eligible individuals who enrolled in the study
Implementation and adaptation of intervention	Research team meeting notes Research coordinator notes Interviews with intervention team and program managers Notes from Community Advisory Board meetings and focus groups	The Consolidated Framework for Implementation Research (CFIR) [91] and Normalization Process Theory [92] will be used to guide the development of interview and focus group guides and CFIR will serve as a framework for data coding and analysis. Directed content analysis will be conducted [93].
Sustainability and scalability	Research team meeting notes Research coordinator notes Interviews with intervention team and program managers Notes from Community Advisory Board meetings and focus groups Notes from Steering Committee	Content analysis [93] will be used to identify themes related to the sustainability and scalability of the intervention [94, 95]

**Table 3:** ACHRU-CPP Intervention Fidelity Guide

Program Components	Data Source(s)
*Staffing and Supervision*	
• Intervention team receives training targeted to learning need	Record of attendance Learning needs assessment
• Monthly outreach meetings between the intervention team and investigators	Meeting notes
*Home visits (in-home, telephone or videoconference)*	
• Up to 3 home/telephone/videoconference visits conducted by the Registered Nurse/Registered Dietitian/Nutritionist	Home visit record
• Screening for diabetes-related complications and comorbidities • Review of medications	Home visit record Standardized clinical assessment tools Medication record Alerts (e.g., medication, diabetes complications)
• Assessment of self-management of diabetes and other chronic conditions and identification of client’s needs and goals • Assessment of caregiver needs (if applicable) • Motivational interviewing to foster behavioural change and encourage self-care	Home visit record My action plan Client-centred care plan Caregiver Strain Index
*Monthly Group Wellness Sessions*	
• Attend up to 6 monthly sessions led by the intervention team • Individual consultations with community program coordinator	Group wellness session record Record of individual consultations with community program coordinator
• Provision of transportation (if needed)	Group wellness session record
*Care Coordination and System Navigation*	
• Registered Nurse helps clients access supports and services in the community as needed	Client-centred care plan
*Monthly Case Conferences*	
• Intervention team attends monthly case conferences	Case conference meeting record
• Intervention team develops and re-evaluates a coordinated care plan for each participant	Client-centred care plan

Interview and focus group data collection guides for clients, caregivers, intervention team members and public and community research partners (available in Additional Files 1, 2, 3 and 4) were developed to assess perceptions of the acceptability, feasibility, implementation, perceived impact, and scalability of the intervention. Constructs from the Normalization Process Theory (NPT) [92] and the Consolidated Framework for Implementation Research (CFIR) [91] were used to develop questions in the interview and focus group guides. Focus groups and interviews with the intervention team and their managers will be held approximately 3 months after the start of the intervention and at the conclusion of the intervention. All members of the intervention team and their managers will be invited to attend the focus groups. In interviews and focus groups, public and community partners will be asked questions about implementation of the intervention based on the CFIR [91] framework as well as questions about their experiences with patient engagement in the study.

#### Adaptation and implementation of the intervention

We will use the framework developed by Stirman and colleagues [96] for reporting adaptations and modifications to evidence-based interventions to track the adaptations made to the intervention over the study period. These will include a description of when and how the adaptations occurred, whether they were planned or unplanned, how fidelity of the intervention was maintained while adapting the intervention, and reasons for and impact of adaptations.

Several data sources will be used to assess implementation outcomes and evaluate how the intervention was implemented and adapted in each of the study sites. These will include: (a) notes of meetings between the intervention team and researchers over the course of the study; (b) transcripts of focus groups and interviews with the intervention team and managers; (c) notes made by the RC and research team members over the course of the study; and (d) notes from the CAB meetings and focus groups with CAB members.

#### Analysis: implementation evaluation

##### Quantitative Data

Descriptive statistics will be used to summarize data related to the adoption of the intervention (i.e., engagement rate, dose of intervention) and the level of fidelity to treatment for each core component of the intervention at 6 months. A fidelity guide (See Table 3) will be used to assess the degree to which the following intervention components are addressed: (a) staffing and supervision (e.g., interventionists receive training and meet monthly with the investigators); (b) home/telephone visits (e.g., up to 3 visits conducted, review of medications, assessment of self-management of diabetes); (c) monthly group wellness sessions (e.g., participants attend up to 6 sessions); (d) care coordination and communication with primary care (e.g., RN helps clients access community supports and services); and (e) monthly case conferences (e.g., interventionists attend monthly case conferences; development of care plan for each client). Frequency and percentages will be used for categorical outcomes and means and standard deviations will be used for continuous outcomes.

##### Qualitative data

All interview and focus group data will be transcribed verbatim, cleaned, and entered into a qualitative data software program (NVivo Version 12, QSR). Directed content analysis [93] will be used to identify codes and themes in the data. The CFIR [91] will be used as a guiding framework for coding the interview data related to implementation. Interviews and focus group data related to the public and community partner engagement strategy will be analyzed inductively to explore both implementation and impacts of engagement (e.g., personal, professional, and research impacts). Two research team members will meet to develop the initial coding structure based on the theoretical frameworks used to develop interview questions (e.g., NPT, CFIR) and review early transcripts and notes from each data source, and the remaining data will be coded using the revised coding framework. All researchers involved in qualitative data analysis will meet regularly to review the coding framework, interim codes and themes, and reach consensus on the final themes.

### Scalability outcomes, measures, and analysis

#### Rationale for designing the trial with scale-up in mind

Planning for scale-up should be part of study design and implementation for many reasons [34]. First, scaling-up often requires changes to areas such as policies, protocols and systems. These changes take time to implement; therefore, planning for needed changes should start as early as possible. Second, supports available in trials are usually more than what can be sustained when interventions are taken to scale, so mechanisms to sustain the intervention need to be developed during study implementation to enable scale-up. Third, there are significant pressures to scale-up interventions like the ACHRU-CPP where existing evidence is promising, particularly given the high number of interventions scaled-up in the absence of any evidence [97]. In a systematic review on scaling-up primary care programs, authors noted vast inconsistencies in scaling-up reports, with none reporting information on assessing scalability [33]. Therefore, experts have stressed the need to use an incremental approach to scale-up with ongoing monitoring of implementation and effectiveness to build a scale-up evidence base [34].

We will conduct a scalability assessment as part of this study, to determine the potential for scale-up of the ACHRU-CPP intervention. The scalability assessment aims to help build the evidence base for the intervention and provide policy-makers and providers with useful tools and resources to serve as the foundation for scale-up planning after the trial ends.

#### Scalability outcomes and measures

A scalability assessment is the first step in scaling up interventions at the population level [94]. The Intervention Scalability Assessment Tool (ISAT) [95] will be used to guide the scalability assessment. Pilot testing of the ISAT has indicated that this tool is perceived as useful to assess scalability and can be applied to a variety of real-world health interventions [98]. ISAT addresses the following outcomes (called domains): Part A describes the problem, intervention, political context, effectiveness and costs/benefits; Part B considers intervention fidelity, adaptations, reach, acceptability, implementation infrastructure (e.g., provider expertise required to deliver the intervention), and sustainability; and Part C is a summative assessment based on questions from Parts A and B that supports a final recommendation on the suitability of the intervention for scale-up [95]. A number of these outcomes (domains) and related measures were discussed above (e.g., intervention, effectiveness, costs, fidelity, adaptations, acceptability) and are summarized using a range of quantitative and qualitative data.

Data sources will include the following: (a) results of the original feasibility study [30], results of the previous trial [31, 32]; (b) results of the effectiveness of the proposed trial; (c) post-intervention interviews with clients and caregivers at each site; (d) focus groups and interviews with intervention team providers and program managers from each site, and (e) notes from meetings of the intervention team, CABs and the Steering Committee.

#### Analysis: scalability assessment

The ISAT [95] consists of items to be considered for each of the domains covered in Parts A and B, and at the end of each domain there are several questions rated on a scale from 0 to 3 representing the readiness assessment of that domain. Part C combines the results of the assessments for each domain and displays the results in the form of a spider web plot, which summarizes the strengths and weaknesses of the intervention and enables comparison across the domains [95]. We will use the ISAT to create spider web plots summarizing the readiness for scale-up at each site.

The scalability assessment, as with scale-up itself, requires collaboration and the collective effort of multiple stakeholders. Therefore, a team approach will be used to complete the ISAT, with the team consisting of the researchers, intervention team, managers, and members of the CABs and Steering Committee. Difficult issues will need to be considered during this process, such as when sufficient evidence exists to determine the suitability for scale-up, how the intervention can be adapted to diverse settings while maintaining fidelity, how the intervention can become sustainable over the medium- and long-term, where scale-up will begin, and important strategic/political/environmental contextual factors that influence scale-up.

### Other outcomes, measures and analyses

#### Intervention team outcomes and measure

To better understand the impact of the intervention on interprofessional team function, the Partnership Self-Assessment Tool (PSAT) [77] will be administered to the intervention team (i.e., RN, RD/Nutritionist, PC, and their managers) near the start and end of the intervention at each site. The PSAT includes items on synergy, leadership, efficiency, administration and management, resources, decision making, benefits and drawbacks of partnership and satisfaction with the partnership.

#### Public and community partner outcome and measures

The public and community partner engagement strategy will be evaluated using surveys completed by members of the CABs and patient/caregiver research partners: (a) the Quality Involvement Measure [78]; (b) the Patient-Centred Outcomes Research Institute’s Ways of Engaging-Engagement Activity Tool (WE-ENACT) Patient/Stakeholder 2.0 self-report tool [79]; and (c) selected items from the Patient-Centred Outcomes Research Institute’s Engagement Activity Inventory NET-ENACT tool [80]. CAB members will also be invited to participate in focus groups at two time points (e.g., near the start and the end of implementation in each site) to explore perspectives on study engagement strategies. Similarly, co-investigator patient partners will be invited to participate in individual interviews for their views on the engagement strategy.

#### Analysis: other outcomes evaluation

We anticipate that small sample sizes will limit the ability to conduct inferential statistical analyses of the data from the PSAT and surveys completed by the public and community partners. Therefore, descriptive statistical analyses will be used to summarize the key responses, patterns and trends evident in these survey data.

### Ethics

The study is being conducted in accordance with the Tri-Council Policy Statement, Ethical Conduct for Research Involving Humans [99]. Institutional ethics approval was obtained from the following: the Hamilton Integrated Research Ethics Board (#5101); the Scarborough Health Network Research Ethics Board (#NEP-18-014); the Unity Health Toronto Research Ethics Board (#18-336); University of Prince Edward Island Research Ethics Board (#6008019); Prince Edward Island Research Ethics Board; and Centre intégré universitaire de santé et de services sociaux (CIUSSS) de la Capitale-Nationale (MP-13-2019-1670). Ethics approval will be renewed on an annual basis as required for the study duration. Informed consent will be obtained from participants (older adults, caregivers, providers, managers, public and community partners) by the RA before study enrolment.

### Modifications triggered by COVID-19 pandemic

Below we discuss the details of all pandemic-related impacts and changes made to the protocol to address/mitigate them.

#### Overview of pandemic impact, importance and key decisions

The trial was underway at all sites at the time that the COVID-19 pandemic hit Canada (March 2020). As noted in the CONSERVE guidelines, the impact of the pandemic on studies already underway “is an exemplar” of extenuating circumstances that are unavoidable and beyond the control of the investigators, funding agencies, and trial sites [58]. In our study, the pandemic resulted in a range of impacts at the sites, including delays in the implementation of the intervention, discontinuation of the intervention, and shifting from in-person to virtual delivery of the intervention and data collection methods. These impacts in turn triggered important modifications to the study protocol that had implications for the study feasibility, intervention implementation, intervention effectiveness, and analytical methods in the trial. The research team planned, reviewed and achieved consensus on the mitigation strategies proposed to address the pandemic impacts.

The pandemic resulted in stopping the study at all sites on March 16, 2020. The next steps at each site were dependent on the stage of the study at the site, with the result being the offering of different doses and/or delivery formats for the intervention to participants within and across sites. We moved to virtual approaches to deliver the intervention and to collect data at all sites, which required obtaining ethics approvals at the sites. One site had the intervention team re-deployed to deal with COVID-19 issues and the intervention could not be restarted. The details of these and other impacts and the mitigation strategy to address them are outlined below for the SPIRIT checklist items that were affected.

#### Trial Registration, protocol version, amendments, research ethics approvals

The shift to virtual delivery of the intervention by videoconference and virtual data collection by telephone resulted in updates to the trial registration, various amendments to the study protocol, and re-submissions to the institutional ethics boards at each site to obtain approval for these changes to the methods.

#### Funding

Delays in implementing the intervention at several sites resulted in extending the study duration past the original deadline outlined in the funding agreements. Fortunately, funding agencies provided a one-year extension to assist with delays resulting from the pandemic.

#### Roles and responsibilities

The roles of the intervention team, RCs and RAs will not change, in that they will still be responsible for delivering the intervention, monitoring trial performance, and collecting data. However, the shift to virtual delivery and data collection requires additional access to secure virtual technology and the ability to use it effectively to deliver care and obtain data. Also, RCs and intervention team members will take on additional responsibilities related to training participants in the use of technology to support the virtual receipt of the intervention. Overall, the intervention team, RCs and RAs are technologically-capable, and investigators will support them as necessary to ensure that any technological challenges will be addressed.

#### Study setting

The trial had to be completely stopped at one of the Quebec sites due to the re-deployment of members of the intervention team to deal with COVID-19 issues. The research team from Prince Edward Island has agreed to recruit an additional 44 participants and provide the intervention virtually by videoconference or by telephone, based on the participant’s preferences, using the same RA, RC and intervention team. Although this reduces the diversity of our sample due to a smaller representation from the province of Quebec, it allows us to preserve the overall sample size of the study.

#### Intervention

The virtual delivery of the home/telephone visits and the group wellness sessions has been proposed to allow the intervention to proceed where the trial was either already underway or about to begin (see *Overview of Pandemic Impact, Importance, and Key Decisions*). The shift to this virtual environment may present challenges to participants, in that they may not be comfortable with videoconferencing technology or have access to it or the Internet/Wi-Fi. For the intervention team, some clinical assessment tools need to be eliminated as they cannot be administered virtually (e.g., frailty assessment, Timed Up and Go (mobility) tests, home environment assessment), which may impact the development of care plans. Further, the monthly team case conferences, originally proposed as in-person team meetings, need to shift to virtual meetings, which may impact team collaboration.

A range of mitigation strategies have been proposed to address these impacts. For the duration of the intervention, tablets and/or Internet/Wi-Fi access will be provided to all participants that require these to enable their virtual participation in the intervention. RCs will train all participants on the use of the tablets and Internet/Wi-Fi as needed. Most clinical assessment tools have remained in place and can be administered virtually, thus the elimination of a select few is not expected to significantly impact the development of a care plan. Maintaining regular case conference schedules as well as increased comfort with online communications are expected to continue to facilitate team collaboration.

#### Participant timeline

Delays in starting and continuing the intervention at some sites resulted in extending the duration of the study past the planned study end dates. Even with the one-year extensions granted by the funding agencies, the planned 12-month follow-up assessment exceeded the end dates of the study at some sites. The mitigation strategy proposed to address this issue was to eliminate the 12-month follow up assessment.

#### Sample size

Sample size will be maintained despite the loss of one of the Quebec sites due to the Prince Edward Island research team offering to recruit an additional 44 participants.

#### Recruitment

Delays in starting the intervention have resulted in recruiting participants at different points in time at the sites, some prior to the pandemic and others during the pandemic. If participants are more recently enrolled at the sites, it is possible that the types of patients or the severity of their diabetes or other chronic conditions may differ from those recruited prior to the pandemic. This has been a concern for acute care sites during the pandemic, but likely less to be an issue in the community-based settings where we are recruiting. Number of chronic conditions is a variable included in our subgroup analyses, which may help in exploring differential effects in participant health status.

#### Data collection methods

All data collection shifted from in-person to virtual methods (phone or video) once the pandemic hit. The collection of data from trial participants by the RAs will be done strictly by telephone. Interview/focus group data from site managers, intervention teams, and CAB members will be done by telephone or videoconference. While this shift triggered a series of changes to the consent forms, no changes were made to the actual data that will be collected.

#### Statistical methods

As noted in *Overview of Pandemic Impact, Importance, and Key Decisions*, decisions on how to proceed with the trial were dependent on the stage of the trial at each site, resulting in variation across intervention participants in terms of the number of home/virtual visits and group wellness sessions that can be offered and the extent to which the intervention components can be delivered virtually versus in person. At all sites, intervention participants will be offered a minimum of one home/virtual visit and one group wellness session. Some clinical assessments were eliminated in the shift to a virtual format, which could result in less information being available to inform the development of a care plan for participants. RAs had to shift to collecting participant data by telephone instead of in person, which could affect the quality of the data collected.

The collective impact of these COVID-related changes on the effectiveness of the intervention is uncertain. Previous trials have not shown a significant dose-response for the intervention, suggesting that a minimum dose of one home/telephone visit and group session may be sufficient [31, 32]. There is some evidence from comparative effectiveness research on similar interventions that outcomes may not change with virtual delivery [100], and other studies cite a range of potential benefits of virtual approaches to diabetes care during the pandemic [101].

We plan to carefully track the type of intervention participants receive and to compare effectiveness and implementation issues for those receiving in-person versus virtual approaches to care. In addition to the pre-planned subgroup analyses noted above, we will conduct a subgroup analysis comparing the effectiveness of the intervention across groups of participants classified according to meaningful differences in exposure to the virtual platform. This subgroup analysis, as with all subgroup analyses, will be regarded as exploratory and offered as relevant background subject to cautious interpretation [85, 87].

#### Consent/assent, informed consent

The shift to virtual delivery of the intervention and data collection triggered changes to the consent forms for trial participants, the intervention team, and CAB members. All changes were made and informed consent was obtained from all participants prior to proceeding with the virtual activity.

## Discussion

This paper describes the design of a multi-jurisdictional pragmatic implementation-effectiveness type II hybrid trial [47] of an intervention (ACHRU-CPP) aimed at improving self-management in older adults with diabetes and MCC. This study will make several important contributions to the existing knowledge base, including:

1. A focus on a population of clinically-complex older adults (with diabetes and MCC), who are often excluded from RCTs.
2. An evaluation of both effectiveness and implementation of the intervention, a hybrid design offering the potential to accelerate the translation of research findings into routine practice [47].
3. Consideration of a large amount of quantitative as well as rich qualitative information about a broad range of outcomes. We have chosen objective, reliable, and valid measures to assess effectiveness and several quantitative and qualitative measures to assess implementation. The study also includes a cost analysis from a societal perspective which has been largely overlooked in previous studies of self-management interventions. Costing data will provide policy- and decision-makers with economic information needed to inform decision making related to integrating the intervention into usual care practice.
4. Evaluation of the effectiveness and implementation of a self-management program before and during the COVID-19 pandemic. The intervention moved from in-person to virtual approaches with the onset of COVID-19. Information on the acceptability and experiences with in-person and virtual approaches to care from both client and provider perspectives will shed light on the strengths and limitations of each approach and may inform future clinical practice where we expect that elements of virtual delivery will remain in the post-pandemic period.
5. A design with early consideration given to scale-up. We will collect data from many sources (e.g., clients, caregivers, providers, public and community research partners, decision-makers) related to the scalability of the intervention and strategies to facilitate successful scale-up.
6. Insights regarding how to optimally engage patient and research partners in the development and implementation of a complex intervention. Active engagement of these groups is consistent with current recommendations for patient-oriented research [102, 103]. In our study, patients, caregivers, and members of the public at each site will be engaged in various ways such as serving as co-investigators, advising on the implementation of the intervention through membership on CABs and the Steering Committee, developing recommendations for sharing study results, and advising on scale-up planning. We will assess their experiences as research partners using various quantitative and qualitative measures.
7. Complexity will be viewed as the intersection of the intervention and the context within which it is implemented, consistent with recent conceptualizations of complexity as it relates to the development and evaluation of healthcare interventions [49].

These contributions will result from a study design offering several strengths. The study evaluates an intervention that has a high potential for adoption and implementation following trial completion, given the strong ‘buy-in’ among both intervention teams and community partners that we have observed in our prior studies. The pragmatic design will help to maximize the applicability of the intervention to the real-world practice context. Key pragmatic features include recruiting participants who are representative of the population presenting in a variety of clinical practice settings, using existing staff in practice settings wherever possible, supporting flexible delivery of the intervention by providers; and using intention-to-treat analysis [61, 104]. Attention to intervention fidelity will ensure consistency and adherence to the intervention protocol, which will support subsequent scale-up initiatives. Key elements of fidelity include standardized training of the healthcare providers, regular meetings between providers and the research team throughout the intervention period, regular review of documents that provide a record of components of the intervention that have been delivered, and detailed recording of adaptations made to the intervention at each site. The inclusion of multiple provinces and sites will enhance the generalizability of results to other Canadian settings, as it reflects some of the diversity of the target population. Finally, a range of measures and procedures have been included in the study design to address various known or anticipated challenges, such as recruitment and retention of a vulnerable target population and extraneous circumstances triggered by the COVID-19 pandemic.

Ultimately, study results will inform practice and policy related to community-based interventions to enhance self-management among older adults with diabetes and MCC. Study results will shed light on who benefits the most from this self-management intervention and what intervention adaptations are needed across multiple sites and diverse settings and provinces to promote successful implementation and the potential for scalability. The use of a pragmatic effectiveness-implementation trial design will enhance the relevance of results for practitioners and policy makers, thereby enhancing the sustainability and scalability of the ACHRU-CPP intervention.

## Supplementary Information

**Additional file 1.** ACHRU-CPP Semi-Structured Post-Intervention Interview Guides: Client and Caregiver Experiences.

**Additional file 2.** ACHRU-CPP Focus Group Guide for Providers.

**Additional file 3.** ACHRU-CPP Individual Interview Guide for Managers.

**Additional file 4.** ACHRU-CPP Focus Group Guide for Public and Community Research Partners (Community Advisory Boards).

## Data Availability

The data for this research consists of questionnaires, interview and focus group transcriptions and meeting notes. Raw data cannot be publicly released due to the risk of compromising participant confidentiality.

## Abbreviations

ACHRU-CPP: Aging Community and Health Research Unit Community Partnership Program
ANCOVA: Analysis of Covariance
CAB: Community Advisory Board
CSI: Modified Caregiver Strain Index
CESD-10: Center for Epidemiological Studies on Depression (10 items)
CFIR: Consolidated Framework for Implementation Research
CONSERVE: CONSORT and SPIRIT Extension for Randomized Controlled Trials Revised in Extenuating Circumstances
CONSORT: Consolidated Standards of Reporting Trials
COVID-19: Coronavirus Disease 2019
DSSI: Duke Social Support Index
ED: Emergency department
GAD-7: Generalized Anxiety Disorder Scale (7 items)
GFR: Glomerular Filtration Rate
HbA1c: Glycated Hemoglobin
HSSUI: Health and Social Services Utilization Inventory
ICEMAN: Instrument for assessing the Credibility of Effect Modification Analyses;
ISAT: Intervention Scalability Assessment Tool
ITT: Intention-to-treat
LDL: Low-Density Lipoproteins
MCC: Multiple chronic conditions
MCS: Mental Component Summary score
NET-ENACT: Patient-Centred Outcomes Research Institute’s Engagement Activity Inventory tool
NPT: Normalization Process Theory
OARS ADL/IADL: Older Americans Resources and Services Multidimensional Functional Assessment Questionnaire Activities of Daily Living/Instrumental Activities of Daily Living
PASE: Physical Activity Scale for the Elderly
PC: Program Coordinator
PCS: Physical Component Summary Score
PRECIS-2: Pragmatic Explanatory Continuum Indicator Summary (version 2)
PROM: Patient-Reported Outcome Measure
PSAT: Partnership Self-Assessment Tool
RA: Research Assistant
RC: Research Coordinator
RCT: Randomized controlled trial
RD: Registered Dietitian
RN: Registered Nurse
SCREEN II-AB: Seniors in the Community: Risk Evaluation for Eating and Nutrition – abbreviated (8-items)
SDSCA: Summary of Diabetes Self-Care Activities
SF-12: Medical Outcomes Study Short-Form Health Survey (12 items)
SPIRIT: Standardized Protocol Items Recommendations for Interventional Trials
SPMSQ: Short Portable Mental Status Questionnaire
SDM: Substitute decision-maker
TIDieR: Template for Intervention Description and Replication checklist
T1: Time 1, Baseline
T2: Time 2, 6-months after baseline
YMCA: Young Men’s Christian Association
WE-ENACT: Patient-Centred Outcomes Research Institute’s Ways of Engaging-Engagement Activity Tool Patient/Stakeholder 2.0 self-report tool.
