# Supplementary material for: The Aging, Community and Health Research Unit Community Partnership Program (ACHRU-CPP) for older adults with diabetes and multiple chronic conditions: study protocol for a randomized controlled trial
Source: BMC Geriatr. 2022 Feb 4;22:99. doi: 10.1186/s12877-021-02651-7 (PMC8814798; doi:10.1186/s12877-021-02651-7)
Supplement: Supplementary file 1 — Additional file 1. ACHRU-CPP Semi-Structured Post-Intervention Interview Guides: Client and Caregiver Experiences. [file 12877_2021_2651_MOESM1_ESM.pdf]

Additional File 1. Semi-Structured Post-Intervention Interview Guides: Older Adult and Caregiver Experiences

Title of Study: Aging, Community and Health Research Unit (ACHRU) Community Partnership Program for Diabetes Self-Management for Older Adults – Canada

Older Adult Participant Post-Intervention Interview Guide

Thank you for agreeing to take part in an interview today. Before we start, I want to remind you that this call will take about one hour and will be audio-recorded.

In the past 6 months, you have received home visits and telephone visits from a registered nurse [name] and/or a registered dietitian. You have also been invited to attend a monthly wellness session at a community site. The purpose of these visits and wellness sessions were to support you in managing your diabetes and other chronic conditions.

I would like to ask you some questions about the home visits and phone calls you received from [name of nurse and name of dietitian], and the wellness sessions you received. Your answers will help us to learn how to better support people who are living with diabetes and other chronic conditions. Your answers will be kept confidential and will not be shared with your healthcare providers. You do not have to answer all of the questions, if you do not want to.

Do you have any questions, or concerns?

Part A: Unmet Needs

The first set of questions that I will ask you are about **your needs.** These needs could be physical, emotional, mental (or psychological), day-to-day basic needs (e.g., food, transportation, financial, housekeeping, personal care) or other needs.

1. What did you need most in the past six months?
2. How, if at all, did [name of nurse and dietitian and community coordinator at the community centre] help to fill these needs?
   a. If need filled by nurse/dietitian/coordinator at the community centre, ask: What did her help mean to you? How did her help make you feel?
3. How, if at all, did the monthly group sessions help to fill these needs?
4. If the need was not filled by anyone, ask, Is this still an important need of yours?

Ⓒ Aging, Community & Health Research Unit, McMaster University. Not for use, publication, citation or distribution without permission of the Aging, Community & Health Research Unit

5. What, if any, (other) needs of yours **have not been met** in the past 6 months? Again, these needs could be physical, emotional, mental (or psychological), day-to-day basic needs (e.g., food, transportation, financial, housekeeping, personal care) or other needs.

Part B: Home Visits and Phone Calls Provided by [name of nurse and dietitian]

Thank-you for your help so far. The next set of questions is about the home visits and phone calls from [name of nurse and dietitian].

6. When [name of nurse and dietitian] **visited you at your home**, what types of things did she do during those visits?
   a. What did this mean to you?
   b. How did it make you feel?
   c. What, if anything, did you find helpful about [name of nurse and dietitian] visits to your home?
   d. What, if any, of <u>your</u> needs were met through home visits from [name of nurse and dietitian]?
   e. What, if anything, was not helpful, or that you did not like, about the home visits?

7. When [name of nurse and dietitian] **called you on the phone**, what types of things did you talk about?
   a. What did this mean to you?
   b. How did it make you feel?
   c. What, if anything, did you find helpful about the phone calls from [name of nurse and dietitian]?
   d. What, if any, of <u>your</u> needs were met through phone calls from [name of nurse and dietitian]?
   e. What, if anything, was not helpful, or that you did not like, about the phone calls?

8. For other people who are living with diabetes and other chronic conditions, would you recommend that a nurse or dietitian, such as [name of nurse and dietitian], visit the person at home, make phone calls or both? Can you tell me why you would recommend this?

Part C: Monthly Wellness Sessions

The next set of questions is about the monthly wellness sessions you attended at [name of site].

1. What types of things did you do at the monthly wellness sessions? How did it make you feel?

2. What, if anything, did you find helpful about the monthly wellness sessions you attended? (probes: education by nurse or dietitian, exercise, meals, peer support)

ⓒ Aging, Community & Health Research Unit, McMaster University. Not for use, publication, citation or distribution without permission of the Aging, Community & Health Research Unit

3. What, if any of your needs, were met through the monthly wellness sessions?

4. What, if anything, was not helpful, or that you did not like, about the monthly wellness sessions, e.g., exercise, meals, education, or accessibility?

Part D: Patient-Reported Experience and Health System Navigation

Thank you for your answers so far. The next set of questions is about your experience with the **care** that you received from [name of nurse and dietitian]. Some of these care questions may or may not be relevant to your care. If the question is not relevant, please let me know and we will go on to the next one.

1. How, if at all, did [name of nurse, dietitian, or community coordinator at the community centre]:
    a. involve you in decisions about your care?
    b. take your preferences (i.e., the way you like things done) into consideration?
    c. respect your values and beliefs?
    d. teach you how to better manage your health?
    e. take steps to improve your mental and emotional well-being?
    f. take steps to improve your physical health?
    g. help you to work through a problem?
    h. discuss your medications with you?
    i. Help you to connect with other community health or social services to help you?

2. To what extent did the nurse and dietitian help to address your needs or the issues that were most important to you?

3. How happy are you with the **overall care** that you received from [name of nurse and dietitian]? Probe: Why do you say this?

4. Is there anything else about your experiences with [name of nurse and dietitian] that you would like to add that we haven't already discussed?

Part E: Patient-Provider Communication (only if time)

Thank you for your help so far. The last set of questions that I will ask you are about **communication.**

1. When [name of nurse and dietitian] was **speaking** to you:
    a. How easy was it for you to **hear** what she was saying?
    b. How easy was it for you to **understand** what she was saying?
    If issues identified, probe: Can you tell me more?

ⓒ Aging, Community & Health Research Unit, McMaster University. Not for use, publication, citation or distribution without permission of the Aging, Community & Health Research Unit

2. How well did [name of nurse and dietitian]:
    a. **listen** to you?
    b. **understand** you?
    c. **answer** your questions?
    d. **provide** information that was helpful to you?
    Probe, if needed: Can you tell me more?

3. Was the information that you were given by [name of nurse and dietitian] and other health professionals about care:
    a. consistent (across individuals)? How did this make you feel?
    b. confusing? How did this make you feel?

4. Is there anything about your communications with [name of nurse and dietitian] that you would like to add that we haven't already discussed?

Thank you again for taking part in this interview. Your input is very helpful.

ⓒ Aging, Community & Health Research Unit, McMaster University. Not for use, publication, citation or distribution without permission of the Aging, Community & Health Research Unit

Caregiver Post-Intervention Interview Guide

Thank you for agreeing to take part in an interview today. Before we start, I want to remind you that this call will take about one hour and will be audio-recorded.

In the past six months, a registered nurse or dietitian has spoken with you by phone and/or has made visits to the home of your relative/friend. The purpose of her visits and phone calls is to support older persons living with diabetes and chronic conditions.

I would like to ask you some questions about the home visits and phone calls made by [name of nurse and dietitian]. Your answers will help us to learn how to better support people living with diabetes and chronic conditions. Your answers will be kept confidential and will not be shared with healthcare providers. You do not have to answer all of my questions, if you do not want to.

Do you have any questions, or concerns?

Part A: Unmet Needs

The first set of questions that I will ask you are about **your needs.** These needs could be physical, emotional, mental (or psychological), day-to-day basic needs (e.g., food, transportation, financial, housekeeping, personal care) or other needs.

1. In the past six months of caring for your family member/friend who has diabetes and other chronic conditions, what were you **most** in need of?

2. How, if at all, did [name of nurse and dietitian] help to fill this need?
    a. If need filled by nurse or dietitian, ask: What did her help mean to you? How did her help make you feel?
    b. If need was not filled by anyone, ask: Is this still an important need of yours?

3. How, if at all, did the monthly wellness sessions at [name of location] help to fill these needs?

4. What, if any, (other) needs of yours **have not been met** in the past six months while caring for your family member or friend living with diabetes and other chronic conditions? Again, these needs could be physical, emotional, mental (or psychological), day-to-day basic needs (e.g., food, transportation, financial, housekeeping, personal care) or other needs.

ⓒ Aging, Community & Health Research Unit, McMaster University. Not for use, publication, citation or distribution without permission of the Aging, Community & Health Research Unit

Part B: Home Visits and Phone Calls Provided by [name of nurse and dietitian]

Thank-you for your help so far. The next set of questions is about the home visits and phone calls from [name of nurse and dietitian].

5. When [name of nurse and dietitian] **visited you at your home,** what types of things did she do during those visits?
    a. What did this mean to you?
    b. How did it make you feel?
    c. What, if anything, did you find **helpful** about the home visits from [name of nurse and dietitian]?
    d. What, if any, of your needs were met through home visits from [name of nurse and dietitian]?
    e. What, if anything, was **not helpful**, or that you did not like, about the home visits?

6. When [name of nurse and dietitian] **called you on the phone,** what types of things did you talk about?
    a. What did this mean to you?
    b. How did it make you feel?
    c. What, if anything, did you find helpful about the phone calls from [name of nurse and dietitian]?
    d. What, if any, of your needs were met through phone calls from [name of nurse and dietitian]?
    e. What, if anything, was not helpful, or that you did not like, about the phone calls?

7. For other people who are caring for older persons living with diabetes and chronic conditions, would you recommend that a nurse or dietitian, such as [name of nurse and dietitian], visit the person at home, make phone calls or both? Can you tell me why you recommend this?

Part C: Monthly Wellness Sessions

The next set of questions is about the monthly wellness sessions you may have attended with your family member/friend at [name of site].

1. What types of things did you do at the monthly wellness sessions? How did it make you feel?

2. What, if anything, did you find helpful about the monthly wellness sessions you attended? (probes: education by nurse or dietitian, exercise, meals, peer support)

ⓒ Aging, Community & Health Research Unit, McMaster University. Not for use, publication, citation or distribution without permission of the Aging, Community & Health Research Unit

3. What, if any of your needs, were met through the monthly wellness sessions?

4. What, if anything, was not helpful, or that you did not like, about the monthly wellness sessions?

Part D: Caregiver-Reported Experience and Health System Navigation

Thank-you for your answers so far. The next set of questions is about <u>your</u> experience with the **care** that <u>you</u> received from [name of nurse and dietitian]. Some of these care questions may or may not be relevant to your care. If the question is not relevant, please let me know and we will go on to the next one.

1. How, if at all, did [name of nurse and dietitian]:
   a. involve you in decisions about your care?
   b. take your preferences (i.e., the way you like things done) into consideration?
   c. respect your values and beliefs?
   d. take steps to improve your mental and emotional well-being?
   e. take steps to improve your physical health?
   f. ensure that the information given to you from other health professionals involved in your care were consistent (or the same)?
   g. teach you how to better manage your health?
   h. help you to work through a problem?
   i. Help you to connect with other community health or social services to help you?

2. To what extent did the nurse and dietitian help to address your needs or the issues that were most important to you?

3. How happy are you with the **overall care** that you received from [name of nurse and dietitian]? Probe: Why do you say this?

   Is there anything about your experiences with [name of nurse and dietitian] that you would like to add that we haven't already discussed?

ⓒ Aging, Community & Health Research Unit, McMaster University. Not for use, publication, citation or distribution without permission of the Aging, Community & Health Research Unit

Part E: Caregiver-Provider Communication (only if time)

Thank you for your help so far. The last set of questions that I will ask you are about **communication.**

1. When [name of nurse and dietitian] was **speaking** to you:
    a. How easy was it for you to **hear** what she was saying?

    b. How easy was it for you to **understand** what she was saying?
    If issues identified, probe: Can you tell me more?

2. How well did [name of nurse and dietitian]:
    a. **listen** to you?
    b. **understand** you?
    c. **answer** your questions?
    d. **provide** information that was helpful to you?
    Probe, if needed: Can you tell me more?

3. Was the information given by [name of nurse and dietitian] and other health professionals about care:
    a. consistent (across individuals)? How did this make you feel?
    b. confusing? How did this make you feel?

4. Is there anything about your communications with [name of nurse and dietitian] that you would like to add that we haven't already discussed?

Thank you again for taking part in this interview. Your input is very helpful.

ⓒ Aging, Community & Health Research Unit, McMaster University. Not for use, publication, citation or distribution without permission of the Aging, Community & Health Research Unit
