# Supplementary material for: The Aging, Community and Health Research Unit Community Partnership Program (ACHRU-CPP) for older adults with diabetes and multiple chronic conditions: study protocol for a randomized controlled trial
Source: BMC Geriatr. 2022 Feb 4;22:99. doi: 10.1186/s12877-021-02651-7 (PMC8814798; doi:10.1186/s12877-021-02651-7)
Supplement: Supplementary file 2 — Additional file 2. ACHRU-CPP Focus Group Guide for Providers. [file 12877_2021_2651_MOESM2_ESM.pdf]

Additional File 2. Focus Group Guide for Providers

Title of Study: Aging, Community and Health Research Unit (ACHRU) Community Partnership Program for Diabetes Self-Management for Older Adults – Canada

Thank you for taking the time to participate in our focus group. This focus group will be audio-recorded and will take approximately one hour.

I want to start by giving you a bit of an introduction to what we will be talking about today. Today we're going to be talking about the approaches used to plan and conduct the Aging, Community and Health Research Unit's Diabetes – Community Partnership Program. When the questions refer to **the program**, we are referring to the Aging Community and Health Research Unit's Diabetes – Community Partnership Program.

We are interested in how you understand the program, if and how it fits and or has been adapted to meet the needs of your community; leadership, resources and partnerships to support the program and its implementation; as well as its evaluation and impacts. We are also interested in your thoughts about what would need to happen to continue the program after the study is complete.

Making sense of the program

1. *What is your understanding of where the Diabetes – Community Partnership Program (henceforth called the program) came from and why it is being implemented in your community?

2. *How does the program compare to similar existing programs in your community?

3. How complicated is the program? (**Probes**: consider the program's: duration, scope, intricacy and number of steps involved, and whether the program reflects a clear departure from previous practices.)

4. *What advantages/disadvantages do you think the program has compared to existing programs?

Program Fit and Adaptations to the program

5. *How does implementation of the program align with your organization's goals?

6. *How well does the program fit with existing work processes and practices in your setting?

ⓒ Aging, Community & Health Research Unit, McMaster University. Not for use, publication, citation or distribution without permission of the Aging, Community & Health Research Unit

7. Have the norms, values and operational culture of your organization (the implementing agency) been taken into account in the design of the program? How so?

8. *What kinds of changes or alterations have been made to the program to help it to work effectively in your community? (**Probes**: policies, procedures?)

9. *What are issues or complications that have arisen or may arise in integrating the program into your current processes? (**Probes**: How will the program interact or conflict with current programs or processes? Will the program replace or compliment a current program or process? In what ways?)

10. Based on your understanding, can your organization absorb this new program without negative impacts on other programs and services? How so?

Preparation/ Training for program delivery

11. *How well did the training prepare you to deliver the program? (**Probes**: Are there things that you wish we could have talked about more during the training? Is there anything that we should clarify or include in training for others in the future?)

12. What is your perception of the supporting materials to help you understand the program for implementation? Why? (**Probes**: How helpful are the resources (online toolkit, etc) to help you implement and use the intervention?)

13. *How, if at all, have the monthly outreach meetings prepared you for delivering the program?

14. Are there ways that the capacity of your organization has been strengthened through training and support by the research team? Are there ways that capacity could be better supported?

Implementing the program

15. There are six main components in the program including: 1) home visits 2) group wellness sessions 3) monthly team case conferences 4) caregiver support 5) collaboration with the primary care interprofessional team and other specialists and 6) nurse-led coordination and system navigation? What has worked and not worked well in integrating each of these components in the program?

ⓒ Aging, Community & Health Research Unit, McMaster University. Not for use, publication, citation or distribution without permission of the Aging, Community & Health Research Unit

16. *The program is delivered through a team effort. What's working well? What can be improved in working with the team?

17. *What strategies have you used to help older adults manage chronic conditions (please tell me about both successes and challenges.)

18. *How are you integrating considerations of multiple chronic conditions, not just diabetes, in providing care?

19. *Can you provide some examples of how you have integrated self-management; health promotion; system navigation in your care?

Patient needs and experiences

20. *How essential is the program to meet the needs of the individuals served by your organization? (Probes: Is there a perceived need for the program? To what extent do current programs fail to meet existing needs? Will the program meet these needs? How will it fill current gaps?)

21. How do you think the individuals served by your organization will respond to the program?

22. *In what ways will the program meet patient needs, if at all? (**Probes**: Help with self-management? More focus on health promotion? A more holistic approach to care? Better links to community services?)

23. *What barriers do you think the individuals served by your organization have faced in participating in the program? What enables them to participate?

Leadership

24. Are there individuals within the organizations involved who are advocates/champions of the new program? Who are they?

25. *What kind of supports has leadership given you for this program so far and what impact has this had? (**Probes**: What level of involvement has leadership at your organization had so far with the intervention? How do attitudes of different leaders vary? What kind of support or barriers can you expect going forward? Can you provide specific examples? What types of barriers might they create?)

ⓒ Aging, Community & Health Research Unit, McMaster University. Not for use, publication, citation or distribution without permission of the Aging, Community & Health Research Unit

26. What are other influential individuals saying about the program and who are they?

Resources

27. To what degree does your organization have capacity to support the program (**Probes**: technical skills, training, supervision, leadership/coordination, monitoring/evaluation, facilities, human resources, and policies)?

28. *Do you have sufficient resources to implement the program? How so? (**Prompts**: What resources were you counting on? Are there any other resources that you received, or would have liked to receive?)

Partnerships

29. *How have others outside of your organization helped you in implementing the program, if at all? Who are they and what was there role?

Evaluation

30. *How, if at all, does the project design include ways to review progress and incorporate new learning into the implementation process? How has that worked for you?

Outcomes and Impacts

31. What impacts do you think the new program has had on:
    - older adults;
    - family caregivers;
    - providers; and
    - organizations in your community?

Other

32. Is there anything else that you think would be important for us to know about the program and its implementation?

Thank you for taking part in this interview. We really value your perspective and appreciate the ideas you have shared today.

ⓒ Aging, Community & Health Research Unit, McMaster University. Not for use, publication, citation or distribution without permission of the Aging, Community & Health Research Unit
