# Supplementary material for: The Aging, Community and Health Research Unit Community Partnership Program (ACHRU-CPP) for older adults with diabetes and multiple chronic conditions: study protocol for a randomized controlled trial
Source: BMC Geriatr. 2022 Feb 4;22:99. doi: 10.1186/s12877-021-02651-7 (PMC8814798; doi:10.1186/s12877-021-02651-7)
Supplement: Supplementary file 3 — Additional file 3. ACHRU-CPP Individual Interview Guide for Managers. [file 12877_2021_2651_MOESM3_ESM.pdf]

Additional File 3. Individual Interview Guide for Managers

Title of Study: Aging, Community and Health Research Unit (ACHRU) Community Partnership Program for Diabetes Self-Management for Older Adults – Canada

Thank you for taking the time to participate in this interview. This interview will be audio-recorded and will take approximately one hour.

Today we're going to be talking about the approaches used to plan and conduct the Aging Community and Health Research Unit's Diabetes – Community Partnership Program. When the questions refer to **the program**, we are referring to the Aging Community and Health Research Unit's Diabetes – Community Partnership Program.

We are interested in how you understand the program, if and how it fits and or has been adapted to meet the needs of your community; leadership, resources and partnerships to support the program and its implementation; as well as its evaluation and impacts. We are also interested in your thoughts about what would need to happen to continue the program after the study is complete.

Making sense of the program

1. * What is your understanding of where the Diabetes – Community Partnership Program (henceforth called the program) came from and why it is being implemented in your community? (Probe: are you aware of any evidence that shows whether or not the program will work?)

2. *How does the program compare to similar existing programs in your community?

3. How complicated is the program? (**Probes**: consider the program's: duration, scope, intricacy and number of steps involved, and whether the program reflects a clear departure from previous practices.)

4. *What advantages/disadvantages do you think the program has compared to existing programs?

Program fit and adaptations to the program

5. *How does implementation of the program align with your organization's goals?

6. *How essential is this intervention to meet the needs of the individuals served by your organization or your organization's goals and objectives?

ⓒ Aging, Community & Health Research Unit, McMaster University. Not for use, publication, citation or distribution without permission of the Aging, Community & Health Research Unit

7. *What kinds of high-priority initiatives or activities are already happening in your setting? (Prompts: What is the priority of getting the program implemented relative to other initiatives that are happening now? Does the implementation conflict with these priorities? Does the implementation help achieve (or relieve pressure related to) these priorities?

8. *Have the opportunities and constraints of the political, policy, health-sector and other institutional factors been considered in designing the program for your setting?

9. Have the norms, values and operational culture of your organization (the implementing agency) been taken into account in the design of the program? How so?

10. *How well does the program fit with existing work processes and practices in your setting?

11. *What kinds of changes or alterations have been made to the program to help it to work effectively in your community? (**Probes**: policies, procedures?)

12. *What are issues or complications that have arisen or may arise in integrating the program into your current processes? (**Probes**: How will the program interact or conflict with current programs or processes? Will the program replace or complement a current program or process? In what ways?)

13. *Based on your understanding, can your organization absorb this new program without negative impacts on other programs and services? How so?

Patient needs and experiences

14. *How essential is the program to meet the needs of the individuals served by your organization? (Probes: Is there a perceived need for the program? To what extent do current programs fail to meet existing needs? Will the program meet these needs? How will it fill current gaps?)

15. How do you think the individuals served by your organization will respond to the program?

16. *In what ways does the Program meet patient needs, if at all? (**Probes**: Help with self-management? More focus on health promotion? A more holistic approach to care? Better links to community services?)

ⓒ Aging, Community & Health Research Unit, McMaster University. Not for use, publication, citation or distribution without permission of the Aging, Community & Health Research Unit

17. *What barriers do you think the individuals served by your organization have faced in participating in the program? What enables them to participate?

Leadership

18. Are there individuals within the organization who are advocates/champions of the new program? Who are they?

19. *What kind of supports has senior leadership given you for this program so far and what impact has this had? (**Probes**: What level of involvement has leadership at your organization had so far with the intervention? How do attitudes of different leaders vary? What kind of support or barriers can you expect going forward? Can you provide specific examples? What types of barriers might they create?)

20. *What are other influential individuals saying about the program and who are they?

Resources

21. *What costs were incurred to implement the intervention? What costs were considered when deciding to implement? (Time 2: What costs do you anticipate if you are to continue the program beyond the study?)

22. *Do you have sufficient resources to implement and administer the program (*beyond the study* - for Time 2 interview)? How so? (**Probes**: What resources were you counting on? Are there any other resources that you received, or would have liked to receive? Were major additional human or financial resources and commodities needed to introduce the intervention?)

23. To what degree does your organization have capacity to support the program (*beyond the study* - for Time 2 interview) (**Probes**: technical skills, training, supervision, leadership/coordination, monitoring/evaluation, facilities, human resources, and policies)?

24. Are there ways that the capacity of your organization has been strengthened through training or other supports from the research team? Are there ways that capacity could be better supported?

Partnerships

25. *How have others outside of your organization helped you in implementing the program, if at all? Who are they and what was there role?

ⓒ Aging, Community & Health Research Unit, McMaster University. Not for use, publication, citation or distribution without permission of the Aging, Community & Health Research Unit

Evaluation

26. *How, if at all, does the project design include ways to review progress and incorporate new learning into the implementation process?

Outcomes and Impacts

27. *What impacts do you think the Community Partnership Program has had on:
    - older adults
    - family caregivers,
    - providers, and
    - organizations in your community?

Other

28. Do you have any additional comments or feedback on the Diabetes – Community Partnership Program study that you would like to share?

Thank you for taking part in this interview. We really value your perspective and appreciate the ideas you have shared today.

ⓒ Aging, Community & Health Research Unit, McMaster University. Not for use, publication, citation or distribution without permission of the Aging, Community & Health Research Unit
