# Supplementary material for: The Aging, Community and Health Research Unit Community Partnership Program (ACHRU-CPP) for older adults with diabetes and multiple chronic conditions: study protocol for a randomized controlled trial
Source: BMC Geriatr. 2022 Feb 4;22:99. doi: 10.1186/s12877-021-02651-7 (PMC8814798; doi:10.1186/s12877-021-02651-7)
Supplement: Supplementary file 4 — Additional file 4. ACHRU-CPP Focus Group Guide for Public and Community Research Partners (Community Advisory Boards). [file 12877_2021_2651_MOESM4_ESM.pdf]

Additional File 4. Focus Group Guide for Public and Community Research Partners (Community Advisory Boards)

Title of Study: Aging, Community and Health Research Unit (ACHRU) Community Partnership Program for Diabetes Self-Management for Older Adults – Canada

Thank you for taking the time to participate in our focus group. This focus group will be audio-recorded and will take approximately one hour.

Today we're going to be talking about the approaches used to plan and conduct the Aging Community and Health Research Unit's Diabetes – Community Partnership Program study. We are interested in how the program has been adapted to meet the needs of communities and what will need to happen to continue the program after the study is complete. We will also be talking about how patients, caregivers, and other community partners have been engaged as partners in the research team.

When we talk about engagement, we're talking about the ways that you have participated or been involved in the work associated with the Diabetes – Community Partnership Program or ways you could be engaged.

When we're talking about research work, we are referring to the many different stages of the Diabetes – Community Partnership Program's work in which you may have participated or been engaged. You may have been involved in:

- developing the partnership
- refining the research proposal
- helping us understand your community
- shaping how the study works, refining recruitment strategies, collecting and analyzing data, developing key messages about the study results, and so on.

With these ideas in mind, we'd like to start with some questions about the Diabetes – Community Partnership Program.

1. What is your understanding of the Diabetes – Community Partnership Program where it came from and why it is being implemented in your community?
   Probe: What kind of information or evidence are you aware of that shows whether or not the Diabetes – Community Partnership Program will work in your community? Probe: How sound is the evidence?

2. How complicated is the Community Partnership Program? Please consider the following aspects of the program: duration, scope, intricacy and number of steps involved, and whether the program reflects a clear departure from previous practices.

ⓒ Aging, Community & Health Research Unit, McMaster University. Not for use, publication, citation or distribution without permission of the Aging, Community & Health Research Unit

3. How does the Community Partnership Program compare to similar existing programs in your community? How will the Community Partnership Program fill current gaps?

    a. What advantages and disadvantages do you think the Community Partnership Program has compared to existing programs?

4. Can you describe how the Community Partnership Program will interact or conflict with current programs or processes?

5. What kinds of changes or alterations do you think have been made to the Community Partnership Program to help it to work effectively in your community? (Probes: policies, procedures?)

6. In what ways will the Community Partnership Program meet patient needs?

7. Are there ways that the program should be altered to meet needs and preferences? (e.g., help with self-management? More focus on health promotion? A more holistic approach to care? Better links to community services?)

8. What barriers do you think older adult study participants in your community will face in participating in the Community Partnership Program?

9. How essential is this intervention to meet the needs of the individuals served by your organization or your organization's goals and objectives?

10. What kinds of high-priority initiatives or activities are already happening in your community?
    a. What is the priority of getting the program implemented relative to other initiatives that are happening now?
    b. Will the implementation conflict with these priorities?
    c. Will the implementation help achieve (or relieve pressure related to) these priorities?

11. Will someone (or a team) outside the organization implementing the program help with implementing the intervention? Who are they and what is there role? How helpful do you think they are/ will be?

12. How does implementation of the intervention align with broader provincial, regional or organizational goals? Probe: Have the opportunities and constraints of the political, policy, health-sector and other institutional factors been considered in designing the project?

ⓒ Aging, Community & Health Research Unit, McMaster University. Not for use, publication, citation or distribution without permission of the Aging, Community & Health Research Unit

Now we'd like to ask you some questions about the processes used to engage patients, caregivers, and other community partners as partners in the Community Partnership Program research team.

13. What have been your experiences of engagement in the Community Partnership Program study to date?
    Probes: What helps you to be involved in the Community Partnership Program study? (e.g., how included in discussions have you felt? how supported have you felt by the team? how respected or valued have you felt as a partner? how involved have you felt in identifying

    problems and gaps, working together to develop solutions, or making decisions? have there been times when you have not felt valued or respected, or not supported by the team?)

14. Can you give any examples of how the study has been changed because of patient, caregiver, and community partner engagement?
    Probe: have the researchers told you that they have changed anything because of what you have said? Have there been any changes in how the Diabetes – Community Partnership Program was adapted, delivered, or evaluated? Have there been any changes in the Diabetes – Community Partnership Program study tools, documents or training/support resources based on your input?

15. In what ways, if any, could the Community Partnership Program study better gain value through the engagement patients, caregivers, and community members?

16. What impact, if any, has there been on you personally or professionally as a result of being involved in the research?

17. Those are all of the questions that we have for you today. Is there anything else that you would like to share about conducting the Community Partnership Program study in your community or the processes used to engage older adults and other community members as partners in the research team?

Thank you for taking part in this interview. We really value your perspective and appreciate the ideas you have shared today.

ⓒ Aging, Community & Health Research Unit, McMaster University. Not for use, publication, citation or distribution without permission of the Aging, Community & Health Research Unit
